# Consolidative Chemoradiotherapy After Induced Chemotherapy Is an Optimal Regimen for Locally Advanced Pancreatic Cancer

**DOI:** 10.3389/fonc.2019.01543

**Published:** 2020-01-21

**Authors:** Lili Wu, Yuhong Zhou, Yue Fan, Shengxiang Rao, Yuan Ji, Jing Sun, Tingting Li, Shisuo Du, Xi Guo, Zhaochong Zeng, Wenhui Lou

**Affiliations:** ^1^Department of Radiotherapy, Zhongshan Hospital Affiliated to Fudan University, Shanghai, China; ^2^Department of Medical Oncology, Zhongshan Hospital Affiliated to Fudan University, Shanghai, China; ^3^Department of Traditional Chinese Medicine, Zhongshan Hospital Affiliated to Fudan University, Shanghai, China; ^4^Department of Radiology, Zhongshan Hospital Affiliated to Fudan University, Shanghai, China; ^5^Department of Pathology, Zhongshan Hospital Affiliated to Fudan University, Shanghai, China; ^6^Department of General Surgery, Zhongshan Hospital Affiliated to Fudan University, Shanghai, China

**Keywords:** locally advanced pancreatic cancer, neoadjuvant treatment, chemoradiotherapy, chemotherapy, multidiciplinary treatment

## Abstract

**Object:** To evaluate the efficacy and tolerability of consolidative chemoradiotherapy (cCRT) after induced chemotherapy (iCT) for locally advanced pancreatic cancer (LAPC).

**Patients and methods:** Patients with LAPC were enrolled from January 2013 to November 2018. In stage one, all patients received iCT. Those without distant metastasis proceeded to stage two, received 50.4 Gy cCRT with S-1 as radiosensitizer. Efficacy and tolerability were evaluated in all patients.

**Results:** Sixty-five patients enrolled into this study and accepted iCT. Eleven (16.9%) patients got early progressions or declined general condition, 1 (1.5%) patient quit the trial after one cycle of iCT. These 12 patients didn't receive cCRT. The remaining 53 (81.5%) patients received cCRT. After cCRT, 4 of 53 (7.5%) patients accepted radical resection. The treatment was well-tolerated. In stage one, neutropenia and thrombocytopenia were the most frequent toxicities, the severe toxicity (grade 3 and 4) were 26.2 and 20.0%, respectively. In stage two, fatigue (45.3%) and nausea (41.5%) were the most frequent toxic effects but most were mild. The median overall survival (OS) of whole group was 18.1 months [95% CI, 15.11–21.03 months]. The OS of patients with early progression and patients accepted cCRT were 7.6 months [95% CI, 5.22–10.02 months] and 19.5 months [95% CI, 18.08–20.95 months], respectively (*P* < 0.001). The PFS of the 53 patients was 10.3 months [95% CI, 8.54–11.96 months] and survival rates at 1- and 2- years were 84.8 and 24.3%, respectively.

**Conclusion:** The current results indicate that iCT is a useful screening method to selecting LAPC patients with less-aggressive biological behavior. cCRT after iCT in patients with LAPC is an optimal treatment. The prognosis of patients who received complete treatment is significantly improved.

## Introduction

Pancreatic ductal adenocarcinoma is one of the most fatal malignant tumors. There were an estimated 90,100 new diagnoses and 79,400 deaths from pancreatic cancer in China in 2015, and the number is still rising (1, 2). Among these about 30% were locally advanced (LAPC) without resectability because of superior mesenteric artery or celiac involvement. The treatment strategy of LAPC patients was similar to metastasis pancreatic cancer in many institutes and the prognosis was dismal, with about 12 months' median survival (3, 4).

The prognosis of LAPC has improved slowly in the past few years, mostly because of the progress in chemotherapy and chemoradiotherapy (CRT). Chemotherapy is the mainstay of the treatment for LAPC. The efficacy of several regimens, like gemcitabine plus oxaliplatin (GEMOX), S-1, gemcitabine plus S-1 (GS), FOLFIRINOX (oxaliplatin, irinotecan, leucovorin, and fluorouracil), nab-paclitaxel plus gemcitabine (AG), and nab-paclitaxel plus S-1 (AS) have been proved and widely used in clinical practice (3–7). The role of CRT is not clear, but consolidative CRT (cCRT) after induction chemotherapy (iCT) has been reported. In a retrospective study of LAPC, Huguet et al. reported that after 3 months of iCT, the addition of cCRT in the patients without metastasis elongated the OS from 11.7 to 15.0 months (*P* = 0.0009) (8). Krishnan et al. reviewed the data of 323 LAPC patients who received CRT in their center from 1993 to 2005, among them 76 were underwent iCT before cCRT and the OS was 11.9 months. In comparison, 247 patients who received CRT directly, the OS was only 8.5 months (*P* < 0.001) (9). These findings suggest that cCRT not only improves local tumor and symptoms control, but also prolongs survival. In addition, after chemotherapy and radiotherapy, some patients may have significant tumor regression and radical surgery would be possible so as to obtain long-term survival.

This is a single-center, prospective study in patients with LAPC. We used iCT at first, followed by cCRT, patients would accept operation if LAPC turned to be resectable, otherwise chemotherapy would be given after cCRT until tumor progression or toxicity appeared. The aim of the study was to test the efficacy of this combination therapy to see whether the OS of LAPC could be improved. Another purpose of this study is to test the feasibility and efficacy of S-1 as a radiosensitizer, which is widely used in Asia countries and the result of JASPAC 01 proved high treatment efficacy in Asian population for pancreatic cancer.

## Patients and Methods

### Patients

Patients with pathological proved LAPC were enrolled from January 2013 to November 2018 in Zhongshan Hospital, Fudan University. The criteria for LAPC were classified as T4N0-1M0 (stage III) according to American Joint Committee on Cancer (AJCC) TNM Staging of Pancreatic cancer (2010).

Eligibility criteria were: (1) LAPC; (2) age 18–75 years; (3) 0–2 ECOG performance status; (4) proven cytological or histological diagnosis of PDAC; (5) adequate cardiac, liver and kidney function and a good bone marrow reserve; (6) no previous abdominal irradiation. (7) No active concomitant malignancy.

Informed consent was obtained from all patients before treatment. The protocol was approved by the Ethical Committees in Zhongshan Hospital (B2012-138) and was registered in the Chinese Clinical Trial Register (ChiCTR-ONC-12003075).

### Treatment

First, all patients received iCT, after at least two cycles of chemotherapy, patients would accept cCRT if there was no distant metastasis developed.

#### Induced Chemotherapy

The iCT regimens were up to investigators' choice, based on the performance status and drug tolerance of different patients. Toxicity was evaluated every cycle and efficacy was evaluated every two cycles to determine the timing of cCRT.

#### Consolidative Chemoradiotherapy

Patients who completed 2–6 cycles of iCT would receive cCRT, which started 2–4 weeks after last dose of chemotherapy. Radiation used 6 MV or 15 MV X-ray beams delivering daily fractions of 180–200 cGy to a total dose of 50–60 Gy in 25–30 fractions using CT-based, three-dimensional conformal radiation therapy (3-DCRT) or intensity-modulated radiation therapy (IMRT). The planning target volume included the tumor mass and regional lymph nodes (>1 cm, peripancreatic, celiac, superior mesenteric, and porta hepatic) with a 0.7–1-cm margin. The local lymph drainage areas were not included. The kidneys, liver, and spinal cord were contoured during the planning process and dose–volume histograms were used to ensure that normal tissue tolerances were not exceeded. The mean dose of live is <23 Gy; no more that 30% of the combined renal volumes received 20 Gy, and the spinal cord received no more than 40 Gy.

S-1 was administered orally 40 mg twice a day in radiation days as radiosensitizer through radiotherapy.

### Toxicity and Efficacy Evaluation

Physical examinations, whole blood cell counts, serum chemical and serum CA 19-9 concentrations analyses were performed before every cycle of chemotherapy (3 weeks). Contrast-enhanced abdominal CT and/or MRI was performed every other cycle of chemotherapy and 3 weeks after cCRT. Tumor response was assessed according to the Response Evaluation Criteria in Solid Tumors (RECIST 1.0). Toxicity was graded according to the Common Toxicity Criteria, version 3.0 (CTCAEv3).

### Data Management and Statistical Analysis

The primary endpoint of the study is overall survival (OS), which is defined from the day of histological diagnosis to the day of death. The second endpoints of this study were progression-free survival (PFS), treatment toxicity, overall response rate, local disease control rate and 1-, 2-year survival rates. The follow-up time was defined from the last day of cCRT to the day of death or the last follow-up.

Data analysis was proceeded by the Statistical Package for the Social Sciences, version 19.0 (SPSS Inc, Chicago, Ill). Results were expressed as median range for continuous variables and number (percentage) for categorical variables. The Kaplan-Meier method is used to evaluate OS and PFS.

## Results

### Patient Characteristics

Sixty-five patients were enrolled. Demographic data of the patients and their primary tumors are listed in Table 1.

**Table 1:** Patients' characteristics of all patients (*n* = 65).

**Characteristics**	***n***	**%**
**Gender (men:women)**	46 (70.8%):19 (29.2%)	
**Median age (range)**	59.6 (41.9–76.2)	
≥70	8	12.3
<70	57	87.7
**PS**		
0–1	49	75.4
2	16	24.6
**Tumor location**		
Head, neck, and uncinatus	37	56.9
Body and tail	27	41.5
Whole pancreas	1	1.5
**Diagnostic mode**		
IOB	27	41.5
EUS-FNA	38	58.5
**CA 19-9**		
Lower than normal ceiling	18	27.7
≤500 U/mL	25	38.5
>500 U/mL	22	33.8

*IOB, intraoperative biopsy; EUS-FNA, endoscopic ultrasound-guided fine-needle aspiration*.

#### Treatments

**Figure 1:**
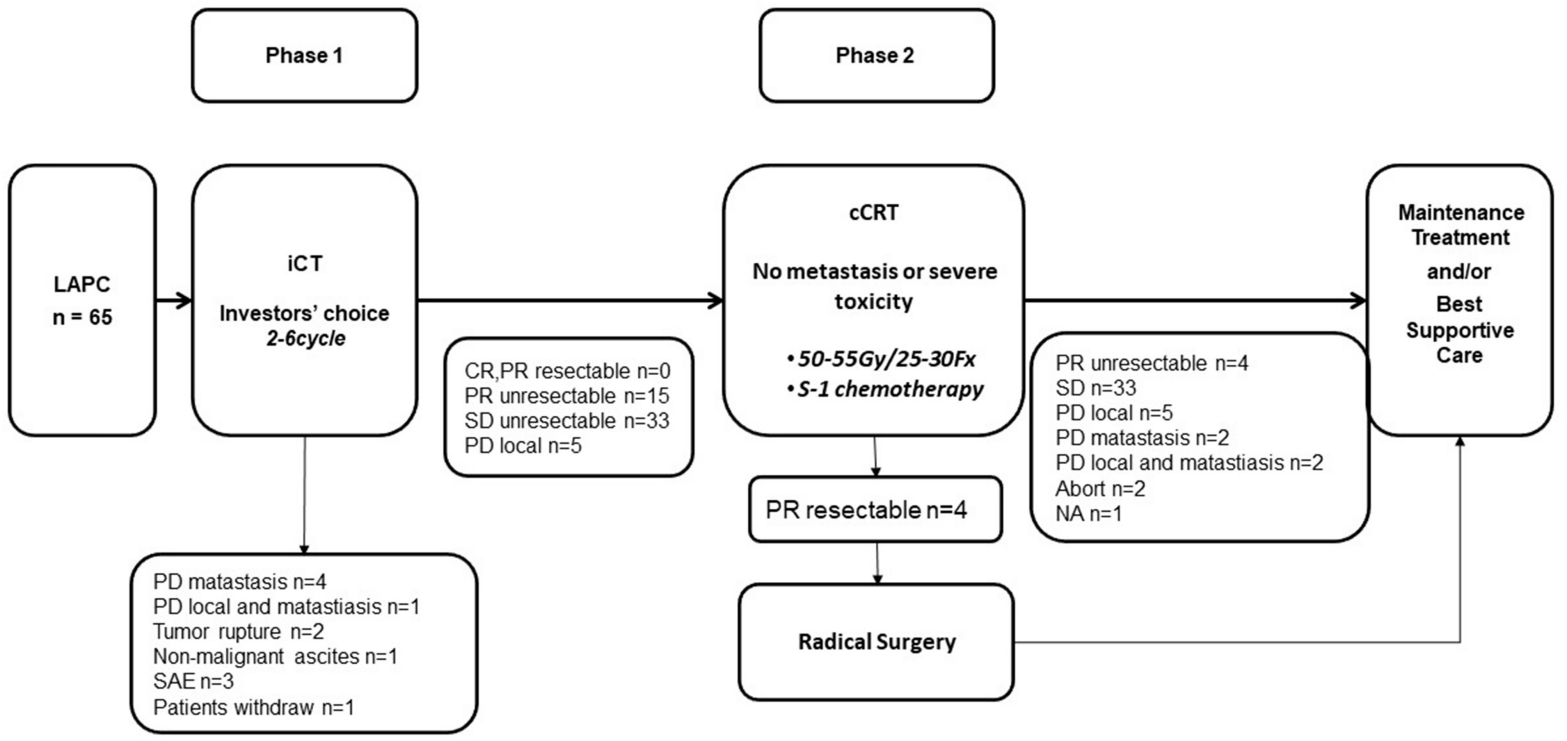
Flowchart.

Treatments according to the protocol are summarized in Figure 1.

1) Induced chemotherapy

Among the 65 patients, 33 patients (50.8%) received S-1 or S-1 based combined chemotherapy (AS), 27 (41.5%) received GEM or GEM-based combined chemotherapy (AG or GEMOX) and five patients (7.7%) received GEM plus S-1 (GS).

In phase 1, five patients developed metastasis, two patients' tumors ruptured, one developed non-malignant ascites. Three patients developed intolerable toxicities and stopped anti-tumor therapy without progression disease. One patient quit this trial. The remaining 53 patients went on to phase 2 and received cCRT.

The detail of iCT was summarized in Table 2.

**Table 2:** Treatment: induced chemotherapy (*n* = 65).

**Induced chemotherapy**	***n***	**%**
S-1 based	33	50.8
GEM based	27	41.5
GS	5	7.7

*GEM, gemcitabine; GS, gemcitabine plus S-1*.

2) cCRT

Characteristics of the 53 patients who received cCRT were outlined in Table 3. Among them, 24 (45.3%) patients had 3-DCRT and 29 (54.7%) had IMRT. One patient aborted cCRT and underwent radical resection after Dt 21.6 Gy/12 Fx. Other 52 patients completed radiotherapy course, the doses were between 50 and 60 Gy (Table 4).

**Table 3:** Patients' characteristics of cCRT (*n* = 53).

**Characteristics**	***n***	**%**
**Gender (men:women)**	38 (71.7%):15 (28.3%)	
**Median age (range)**	59.5 (41.9–76.2)	
≥70	5	9.4
<70	48	90.6
**PS**		
0–1	42	79.2
2	11	20.8
**Tumor Location**		
Head, neck, and uncinatus	27	50.9
Body and tail	25	47.2
Whole pancreas	1	1.9
**Diagnostic mode**		
IOB	22	41.5
EUS-FNA	31	58.5
**CA 19-9**		
Lower than normal ceiling	14	26.4
≤500 U/mL	21	39.6
>500 U/mL	18	34.0

*IOB, intraoperative biopsy; EUS-FNA, endoscopic ultrasound-guided fine-needle aspiration*.

**Table 4:** Treatment: cCRT (*n* = 53).

**Treatment**	***n***	**%**
**Radiotherapy technology**		
3DRT	24	45.3
IMRT	29	54.7
**Radiation dose**		
<50 Gy (abort)	1	1.9
50–55 Gy	49	92.5
56–60 Gy	3	5.7
**Concurrent CT (*****n*** **=** **52)**		
S-1 completed	49	94.2
S-1 abort	3	5.8
**Radical surgery**		
Done	4	7.5
Undone	49	92.5

*cCRT, concurrent chemoradiotherapy*.

Three patients discontinued S-1 after 1- or 2-weeks treatment due to intolerance but all finished radiotherapy.

3) Follow-up treatment

Totally, four of the 53 patients successfully converted to resectable disease. One patient accepted pancreaticoduodenectomy, the postoperative pathology showed complete pathological response. Other three accepted distal pancreatectomy, the tumors showed more than 50% pathological response. All were R0 resections and lymph node negative.

Forty-eight patients continued chemotherapy after completion of radiotherapy. The regimen was determined by the previous treatment reaction and patients' general condition. Thirty-six patients received 2nd-line chemotherapy after tumor progression.

#### Toxicities

The toxicity profile is summarized in Tables 5, 6. The main toxicity of iCT was myelosuppression. The sensory neuropathy mainly occurred in the patients receiving AS and AG chemotherapy. Table 5 shows the details.

**Table 5:** Toxicity of induction chemotherapy (*n* = 65).

**Toxicity**	**Mild (grade 1 and 2)**		**Severe (grade 3 and 4)**		**Totle**	
	***n***	**%**	***n***	**%**	***n***	**%**
**Hematological**						
Neutropenia	15	23.1	17	26.2	32	49.2
Thrombocytopenia	14	21.5	13	20.0	27	41.5
Anemia	13	20.0	4	6.2	17	26.2
**Non-hematological**						
Nausea	17	26.2	6	9.2	23	35.4
Vomiting	10	15.4	3	4.6	13	20.0
Fatigue	12	18.5	8	12.3	20	30.8
Elevated bilirubin	7	10.8	5	7.7	12	18.5
Elevated aminotransferase	15	23.1	7	10.8	22	33.8
Sensory neuropathy	8	12.3	7	10.8	15	23.1
Hand-foot syndrome	5	7.7	3	4.6	8	12.3

**Table 6:** Toxicity of cCRT (*n* = 53).

	**Mild (grade 1 and 2)**		**Severe (grade 3 and 4)**		**Totle**	
	***n***	**%**	***n***	**%**	***n***	**%**
**Hematological**						
Neutropenia	9	17.0	9	17.0	18	34.0
Thrombocytopenia	9	17.0	6	11.3	15	28.3
Anemia	8	15.1	0	0.0	8	15.1
**Non-hematological**						
Nausea	18	34.0	4	7.5	22	41.5
Vomiting	14	26.4	2	3.8	16	30.2
Fatigue	21	39.6	3	5.7	24	45.3
Elevated bilirubin	1	1.9	3	5.7	4	7.5
Elevated aminotransferase	3	5.7	0	0.0	3	5.7
Abdominal pain, distension	17	32.1	1	1.9	18	34.0

During cCRT, myelosuppression was mild, mainly leukopenia (27.9%), non-hematological reactions were in mild to moderate fatigue (39.5%) and nausea (34.9%). See Table 6 for details.

#### Survival and Disease Progression

The median follow-up time is 34.6 months. A total of 60 patients (92.3%) developed tumor progression and 56 (86.2%) died.

The first site of progression was distant metastasis in 14 patients (21.5%), local recurrence in 22 patients (33.8%) and combined local and at distant in eight patients (12.3%). Sixteen patients (24.6%) were found to have uncontrollable elevated CA 19-9 and/or physical status decline, but no radiological evidence of recurrence was obtained.

The PFS was 8.9 months [95% CI, 8.14–9.60 months] in all 65 patients and 10.3 months [95% CI, 8.54–11.96 months] in 53 patients who received cCRT. In the whole group, the 1-year PFS rate was 31.8%.

**Figure 2:**
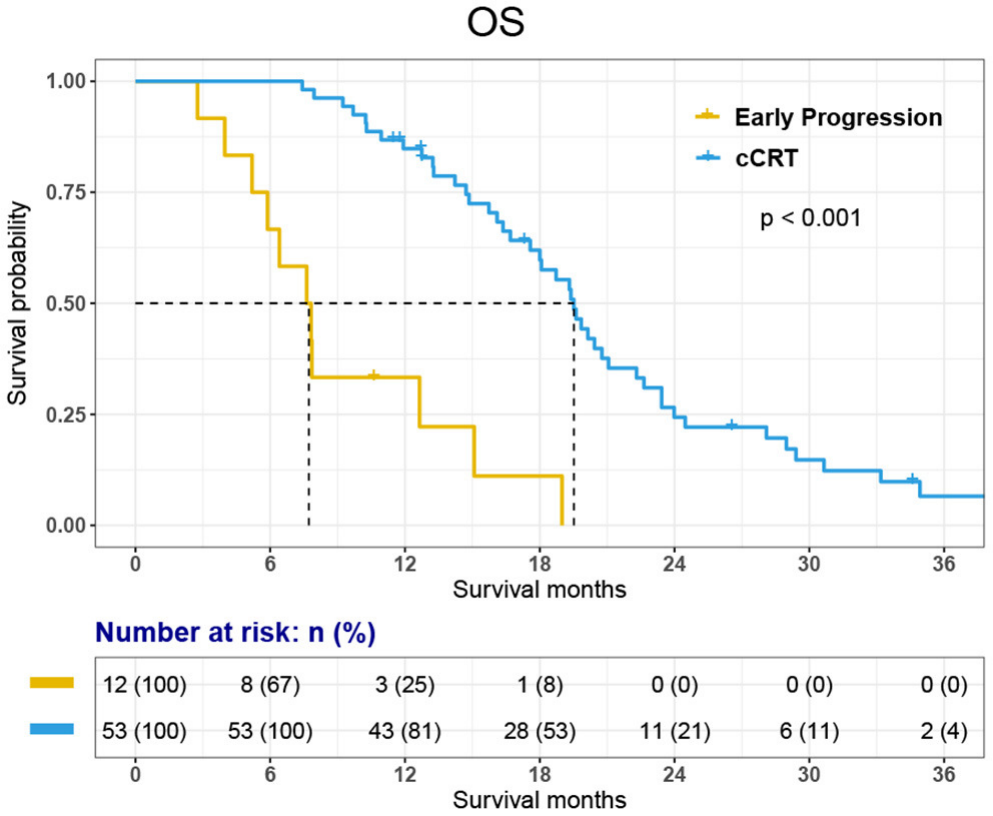
Kaplan-Meier curve for Overall Survival (OS).

**Figure 3:**
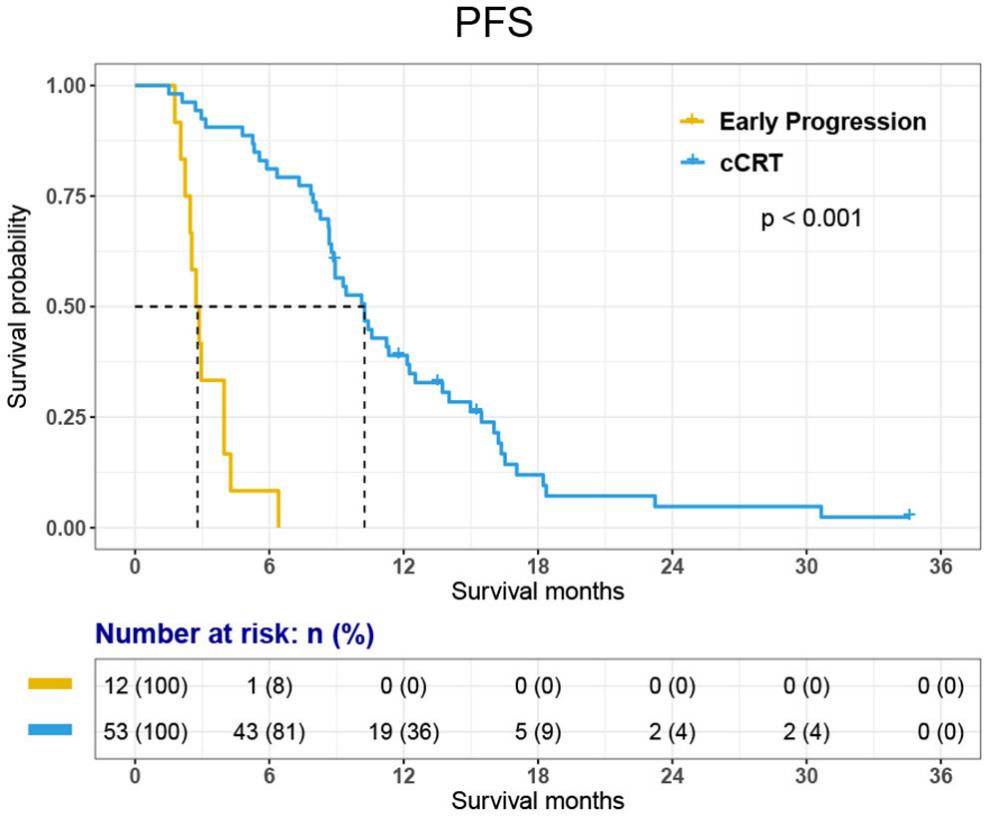
Kaplan-Meier curve for Progression-Free Survival (PFS).

Among the 12 patients who did not receive cCRT, 11 died and 1-year survival was 33.3%. Among 53 patients who received cCRT, 45 died. 1-, 2-, 3-year survival rate was 84.8, 24.3, and 6.6% respectively. The OS of 65 patients was 18.1 months [95% CI, 15.11–21.03 months]. The OS of 53 patients who finished cCRT was 19.5 months [95% CI, 18.08–20.95 months]; while the OS of 12 patients with early progression was 7.6 months [95% CI, 5.22–10.02 months] (Figures 2, 3).

## Discussion

Theoretically, pancreatic cancer is a systemic disease. Chemotherapy is the standard treatment in adjuvant setting and for metastatic pancreatic cancer (14–16). LAPC is not the late-stage, the tumor is locally radiologically aggressive, the invasion of the important arteriovenous structure leading to radical resection difficulties. In addition, because of lacking sensitive examination methods, the possibility of micrometastasis cannot be ruled out (17, 18). In many centers, the treatment strategy of LAPC is equivalent to metastatic pancreatic cancer and utilizing chemotherapy only (3, 4). However, due to the lack of effective treatment options and poor patient tolerance, the prognosis is still poor. For example, FOLFIRINOX and AG are currently considered to be the most effective chemotherapy, and have improved the survival, but the toxicities are also serious. In AG regimen, 1/3 patients need to reduce dosage and more than 70% had a dose delay. About 70% patients have grade ≥ 3 chemotherapy-related bone marrow inhibition.

On the other hand, the value of CRT in LAPC is still controversial because of the inconsistent results of different studies. The main reasons of these different outcomes lie in the discrepancy of radiotherapy target, technique, dosage and radiosensitizer. Fail to select the suitable patients for CRT may also be an influence factor. We enrolled LAPC patients in this study, used iCT as a screening method to select patients with stable disease after 2–6 cycles of chemotherapy so that excluding patients with rapid distant metastasis, which reflected dismal biological behavior of tumor. For patients with early rapid progress, supportive treatment started earlier and no anti-cancer treatment was given to them. In our study, the result revealed the rationality of screening patients, for 53 patients who received cCRT, the OS was as long as 19.5 months. Although this study is not a randomized controlled study, by comparing to the existing literature data, it still shows the effectiveness of this treatment programs.

In recent years, the treatment programs of iCT plus cCRT for LAPC have received widespread attention. Retrospective analysis of big database from different countries and institutes suggested that the strategy of iCT followed with cCRT have a relatively better prognosis (19, 20). In some clinical studies, a certain percentage of T4 patients achieved tumor downstage after neoadjuvant therapy. These patients accepted radical resection and obtained curable possibilities. Ko et al. enrolled 25 LAPC patients and accepted GEM + Cis combination chemotherapy. After iCT, 17 patients did not appear distant metastasis and move to the stage of cCRT. The median survival of 17 patients was 17.0 months compared with 13.6 months of all 25 patients (10). Leone et al. published their prospective study in 2013, the study recruited both locally advanced and borderline resectable pancreatic cancer. Finally, 24 patients with LAPC were enrolled. One case showed early progression and 23 underwent cCRT. Two of the 24 patients accepted radical surgery. The OS was 13.3 months for all 24 patients, higher than expected (11). Passardi et al. published their phase II study results. In their study, 40 patients were enrolled, after four cycles of GEMOX, hypofractionated radiotherapy (35 Gy/7 Fx) were conducted, after that, four additional cycles of GEMOX. The OS of this study is 15.8 months (13). Hammel et al. reported in 2016 the results of the LAP07 study in patients with and without cCRT. In this prospective study, patients accepted Gem and erlotinib induction therapy on stage one, patients without progression were randomly divided into cCRT with Cap and control groups. The cCRT group received a total dose of 54 Gy/30 Fx 3-DCRT, Cap as radiosensitizer. A total of 133 patients in the cCRT group and 136 in the control group were enrolled. The OS of the cCRT group was 15.2 months and the control group was 16.4 months (*P* = 0.8), with no statistical significance. Therefore, they concluded that the value of cCRT after iCT in patients without distant metastasis remains to be studied (12) (Table 7).

**Table 7:** Relative researches.

**References**	**Prospective/retrospective**	**Case no**.	**Induced CT**	**cCRT**	**OS (months)**
Ko et al. (10)	Prospective	17	GEM/cis	50.4 Gy; 5 Fu or Cap	17.0
Leone et al. (11)	Prospective	24	GEMOX	50.4 Gy; GEM	13.3
Hammel et al. (12)	Prospective/control	133/136	GEM ± erlotinib	54 Gy;Cap/-	15.2/16.4 (*P* = 0.8)
Passardi et al. (13)	Prospective	40	GEMOX	35Gy/7 Fx	15.8
This study	Prospective	65	Multiagent CT	50.4;S-1	18.1

Considering the differences of general condition and tolerability, our study adopted several chemotherapy regimens, including combined chemotherapy and single agent chemotherapy. In patients with PS score of 0 or 1, we chose combined chemotherapy in three different combinations (AS, AG, and GEMOX). In patients with PS score of 2, we chose single-agent (GEM or S-1) chemotherapy. This maybe the imperfect part of the study, but during treatment, such treatment options showed good compliance. The cCRT plan is also based on the patient's physical status, tumor size, and adjacent tumor anatomy, given a dose between 50 and 55 Gy. Although this treatment strategy is not uniform enough, it satisfies the real world for different treatment programs fine-tuning. This may also be due to the results of well toleration and longer OS in our treatment.

S-1 is an oral 5-FU prodrug. Several phase II studies of S-1 cCRT have demonstrated an acceptable toxicity profile and promising efficacy with a response rate of 24%-41% and median survival of 12.9–16.8 mouths (21–24). Nowadays, it's widely used in Asia. Instead of using S-1, capecitabine, another oral 5-FU prodrug has been reported in Western countries as a radiosesitizer with a promising result (25, 26). In our study, we used S-1 cCRT in stage two, the efficacy was surprisingly good with mild toxicities.

Although there are many reports on surgical resection of neoadjuvant patients with LAPC, only 4 (6.2%) patients underwent radical pancreasectomy in this study. To review similar clinical trials, the proportion of LAPC patients undergoing radical surgery after induction therapy is relatively low. Laparoscopic exploration showed that LAPC patients have a higher rate of peritoneal implantation, so that the prognosis of LAPC patients is worse than borderline resectable and resectable patients (11). This group of patients requires of experienced doctors to make the right treatment options, adjusting their treatment strategies timely, so that patients at different periods can have individualized treatment and get a better prognosis.

In addition, we found that even with cCRT as a local consolidation therapy, local control is still an important failure mode, suggesting the need for higher doses of radiation and/or stronger synchronized chemotherapy, or more aggressive local surgery. These need to be weighed against treatment safety in future clinical trials.

## Conclusion

The current results indicate that iCT is a useful screening method to selecting LAPC patients with less-aggressive biological behavior. cCRT after iCT in patients with LAPC is a practical and optimal treatment option with reasonable toxicities. The prognosis of patients who received cCRT is significantly improved.

## Data Availability Statement

The datasets generated for this study are available on request to the corresponding author.

## Ethics Statement

The studies involving human participants were reviewed and approved by Ethical Committees of Zhongshan Hospital, Fudan University (B2012-138) and registered in the Chinese Clinical Trial Register (ChiCTR-ONC-12003075). The patients/participants provided their written informed consent to participate in this study.

## Author Contributions

WL contributed to the conception and design of the study, revising the article critically for important intellectual content, and the treatment strategies for each patients. LW, ZZ, and JS contributed to the treatment of concurrent chemoradiotherapy. YZ, YF, LW, and XG took charge of chemotherapy and supportive cares. LW also contributed to follow-ups, gathering data and drafting the article, and analyzed and interpreted data. SR, SD, and LW contributed to efficacy evaluation. YJ contributed to pathological diagnosis. TL contributed to the radiotherapy planning. All authors gave their final approval to the version and WL took the responsibility for submitting the manuscript for publication.

### Conflict of Interest

The authors declare that the research was conducted in the absence of any commercial or financial relationships that could be construed as a potential conflict of interest.
